# Giant Omental Lipoma in a Child

**DOI:** 10.5812/kmp.iranjradiol.17351065.3150

**Published:** 2011-11-25

**Authors:** Vikas Chaudhary, Mahender Kaur Narula, Rama Anand, Isha Gupta, Gurmeen Kaur, Kanika Kalra

**Affiliations:** 1 Department of Radiodiagnosis, Employees’ State Insurance Corporation (ESIC) Model Hospital, Gurgaon, Haryana, India; 2 Department of Radiodiagnosis, Lady Hardinge Medical College, New Delhi, India; 3 Lady Hardinge Medical College, New Delhi, India

**Keywords:** Lipoma, Omentum, Mesentery, Tomography, X-Ray Computed

## Abstract

Omental lipomas are extremely rare tumors of childhood. We report a case of solitary giant lipoma of the omentum in a child, successfully managed by complete excision, without any recurrence on follow-up study.

## 1. Introduction

Omental lipomas are rare benign tumors of infants and children that can be accurately diagnosed preoperatively by ultrasound and abdominal computed tomography (CT). Complete surgical excision is the treatment of choice. We report a case of giant omental lipoma in a 2.5-year-old boy, successfully managed by complete excision without any recurrence on follow-up study.

## 2. Case Presentation

**Figure 1:**
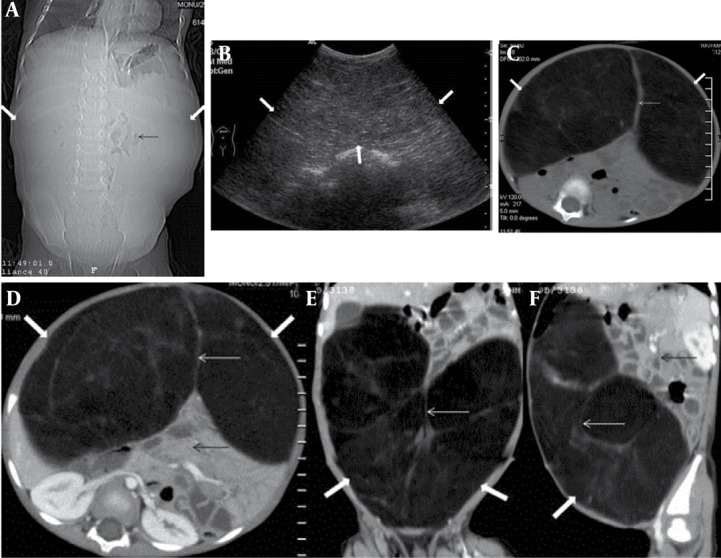
A 2.5-Year-Old Boy Presenting With Complaints of Gradual Abdominal Distention, Intermittent Abdominal Pain and Diarrhea. A, Scannogram of the abdomen shows gross abdominal distension (thick white arrow) and centrally positioned bowel loops (thin black arrow). B, Abdominal ultrasound in the same patient reveals a huge homogeneous echogenic mass (thick white arrow) occupying almost the entire abdomen. C, (Plain) and D, E, F (Contrast-enhanced axial/coronal/sagittal) abdominal CT images of the same patient reveal a well-marginated non-enhancing intraperitoneal encapsulated mass (thick white arrow) of fat density (-70 to -140 HU) with few fibrous septae (thin white arrow) traversing the mass. Bowel loops and retroperitoneal structures are displaced posteriorly (thin black arrow). No inflammatory reaction or infiltration of surrounding tissues is noted.

A 2.5-year-old boy was admitted for evaluation of an abdominal mass. He was a well-developed, well-nourished boy who presented with complaints of gradual abdominal distention, intermittent abdominal pain and diarrhea of two months duration. Palpation of the abdomen revealed a large, elastic mass extending in all the quadrants. Plain X-ray of the abdomen demonstrated gross abdominal distension and centrally positioned bowel loops (Figure 1 A). Ultrasound examination of the abdomen revealed a huge, homogeneous echogenic mass occupying the whole abdomen, displacing the bowel loops and retroperitoneal structures posteriorly (Figure 1 B). The CT revealed a well-encapsulated mass of fat density (-70 to -140 HU). Few thin fibrous septations were also noted within the mass (Figures 1C, D, E & F). However, there was no infiltration of adjacent structures by the mass lesion.A diagnosis of giant omental/mesenteric lipoma was made. Laboratory results were normal. At surgery, it was found to be a large, round mass, arising from the omentum with no adherence to other abdominal organs. Cut section revealed a mass of adipose tissue, which was capsulated and had a smooth glistening white surface. Histological sections showed mature fat. No recurrence was reported on follow-up after 6 months.

## 3. Discussion

Primary tumors of the omentum are very rare. Stout and Cassel described the first reported case of a primary omental tumor in 1942 [1].

Common presenting symptoms of a solid omental tumor include abdominal discomfort (45.5%), abdominal lump (34.9%) and abdominal distention (15.2%). Nausea and weight loss may occur occasionally. Abdominal pain is usually exacerbated in the supine position but relieved in the upright position [1]. The greater omentum is composed mainly of fatty tissue with blood vessels and lymphatics; therefore, a wide range of pathologies have been reported; namely, leiomyosarcoma, fibrosarcoma, hemangiopericytoma, liposarcoma, leiomyoma, lipoma, fibroma and mesothelioma. Lipomas are perhaps the rarest of all the above forms especially the giant ones, described in literature almost exclusively in the form of case reports [2].

In 1980, Giubilei et al. reported a solitary giant lipoma of the omentum and gastrocolic ligament in an 8-year-old boy [3]. In 2005, Luo reported complete excision of a giant omental lipoma in an 11-month-old boy in China [4]. In 2007, a giant omental lipoblastoma was reported in a 10-month-old girl in Spain that composed 20% of her total body weight [5]. Srinivasan et al. reported a case of large omental and mesenteric lipoma in a 9-month-old infant in India which occupied nearly the whole of the abdomen [6]. Another case of a similar huge omental lipoma weighing 12.3 kg in a 13-year-old adolescent girl was reported by Abubakar in 2009 [7].

In conclusion, omental lipoma as other solid abdominal tumors is easily diagnosed by ultrasound (US), but for more precise localization and characterization of the lesion, CT should always be performed.
